# A Report of Two Cases of Malignant Tumor of the Maxillary Sinus Diagnosed Using Fine-Needle Aspiration Cytology

**DOI:** 10.7759/cureus.36506

**Published:** 2023-03-22

**Authors:** Tessei Kuruma, Tetsuya Ogawa, Mariko Arimoto, Kinga Yo, Yasushi Fujimoto

**Affiliations:** 1 Otorhinolaryngology-Head and Neck Surgery, Aichi Medical University Hospital, Nagakute, JPN; 2 Otolaryngology-Head and Neck Surgery, Aichi Medical University Hospital, Nagakute, JPN

**Keywords:** the three-dimensional (3d) ct image, magnetic resonance imaging (mri), computed tomography (ct), cell block method, malignant tumor, maxillary sinus, sinonasal tract, fine needle aspiration cytology (fnac)

## Abstract

Primary and metastatic malignancies arising in the sinuses are rare and histologically diverse. The role of fine-needle aspiration cytology (FNAC) and the cytomorphologic characteristics of these tumors have not been specifically addressed. We described two cases of suspected malignant maxillary sinus tumors in 85- and 90-year-old patients with comorbid conditions, both of whom underwent tissue biopsies that failed to yield a definitive diagnosis. We performed FNAC after imaging confirmed that the malignant tumors were outside the maxillary sinus. The 85- and 90-year-old patients were diagnosed with squamous cell carcinoma (SCC) and adenocarcinoma, respectively. In the latter, the cell block method was used to prepare the specimen, rendering individual cells identifiable. Atypia of the histological structure was confirmed without the influence of cell duplication, a known weakness of FNAC. Thus, the diagnosis was made quickly. We believe that FNAC would be utilized more frequently for the definitive diagnosis of sinonasal tumors as the technique and diagnostic technology improve further.

## Introduction

Primary and metastatic malignancies originating in the sinonasal tract are rare and histologically diverse.

Fine-needle aspiration cytology (FNAC) is accurate, cost-effective, and has quick turnaround times [1]. However, FNAC of sinonasal lesions for the early diagnosis of neoplastic lesions is difficult because of their closed structure; the literature on the role of FNAC in the diagnosis of sinonasal neoplasms is limited [1-3].

Here, we report two cases of maxillary sinus malignancies in which a definitive diagnosis was obtained using FNAC after repeated tissue biopsies failed to yield definitive diagnoses. Tumors were localized using sinus computed tomography (CT) and magnetic resonance imaging (MRI).

## Case presentation

Case 1

**Figure 1:**
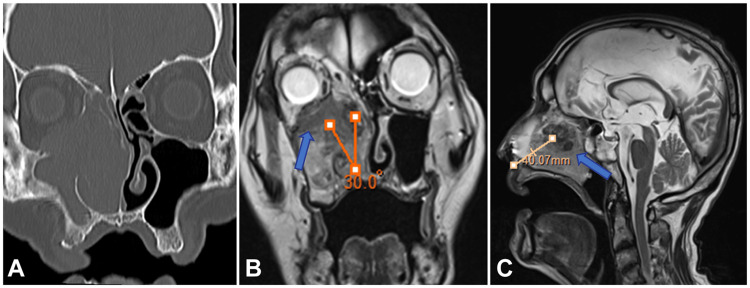
Imaging findings of the paranasal sinuses A) Coronal section sinus CT. B) Coronal T2-weighted MRI image. Low-signal areas indicate areas with suspected malignancy (arrows). The triangular red line indicates the angle from the nasal cavity entrance to the tumor in the medial direction of the orbit. C) Sagittal T2-weighted MRI image. The low-signal area is the site of the suspected malignancy (arrow). The pink line represents the distance from the nasal cavity to the tumor. CT, computed tomography; MRI, magnetic resonance imaging.

An 85-year-old male patient complained of right nasal discharge with an associated transient hemorrhage of three weeks’ duration. An endoscopic examination of the nasal cavity revealed a polypoid mass in the right nasal cavity. CT of the sinus cavity revealed a soft neoplastic shadow filling the right maxillary sinus, with bony destruction of the orbital floor, and infiltration into the orbit, including the inferior rectus muscle (Figure 1A). He had a history of cerebral infarction, chronic obstructive pulmonary disease, and right upper lobectomy for pneumothorax, and was taking an anticoagulant (clopidogrel sulfate) to prevent a recurrence. He had developed dysphagia and gait disturbance, with a decline in activities of daily living (ADL). After the anticoagulants were discontinued, a tissue biopsy was performed on an outpatient basis. A biopsy was performed using a rigid nasal endoscope after superficial anesthesia was administered to the nasal cavity. Two biopsies were performed on a polyp in the right nasal cavity and a tissue biopsy of what appeared to be a mass further back into the nasal cavity; the tissue biopsy results showed a nasal polyp.

The patient was referred to the Department of Otorhinolaryngology at the Aichi Medical University for further examination. Nasal endoscopy revealed a polyp filling the right nasal cavity and a hemorrhagic mass behind the polyp, which was the tumor itself. MRI of the sinus cavity revealed a large mass within the right maxillary sinus protruding into the nasal cavity. The sagittal image demonstrated the presence of a tumor just posterior to the polyp. A tissue biopsy was performed again in the outpatient department.

Before the biopsy, the nasal cavity was superficially anesthetized with gauze, and lidocaine HCI (1%) and epinephrine (1:100,000) were injected topically into the nasal cavity.

Transnasal endoscopic tissue biopsy was performed on the lesion behind the polyp. However, the bulk of the mass could not be biopsied because of excessive bleeding. Histopathological examination revealed an endogenous papilloma with dysplasia, and no atypia, which was cancerous. We considered whether the papilloma had degenerated into a malignant tumor such as a squamous cell carcinoma (SCC).

**Figure 2:**
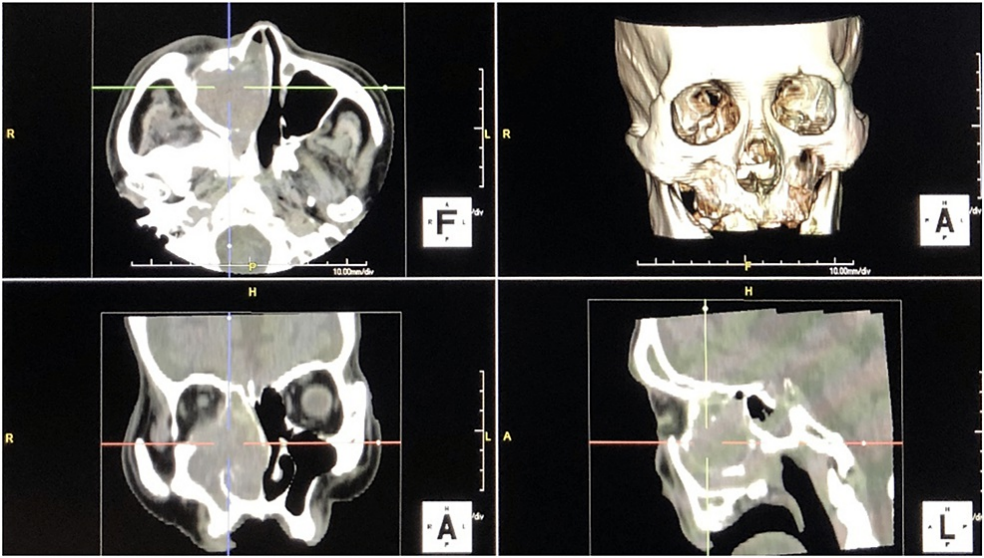
Three-dimensional CT findings of the sinus cavity CT, computed tomography.

A tissue biopsy was performed under general anesthesia. However, because the patient was elderly and had comorbidities, general anesthesia was considered risky. Instead, we performed an FNAC in the outpatient setting. Upon reviewing the sinus MRI, a T2-weighted image showed a high-signal area in the nasal cavity consistent with a polyp. Posterior to that was a slightly low-signal area consistent with a papilloma. Additionally, a low-intensity area bordered by the right orbital floor to the middle nasal meatus was suggestive of a malignant tumor (Figure 1B, 1C). The angle toward the medial right orbit was approximately 30º (Figure 1B). The distance from the nostril was approximately 4 cm on the coronal and sagittal MRI images (Figure 1C). Four sites were sampled for the cytological analysis. The puncture site was imaged three-dimensionally using CT to precisely identify the suspected malignant low-signal area on a T2-weighted MRI (Figure 2).

**Figure 3:**
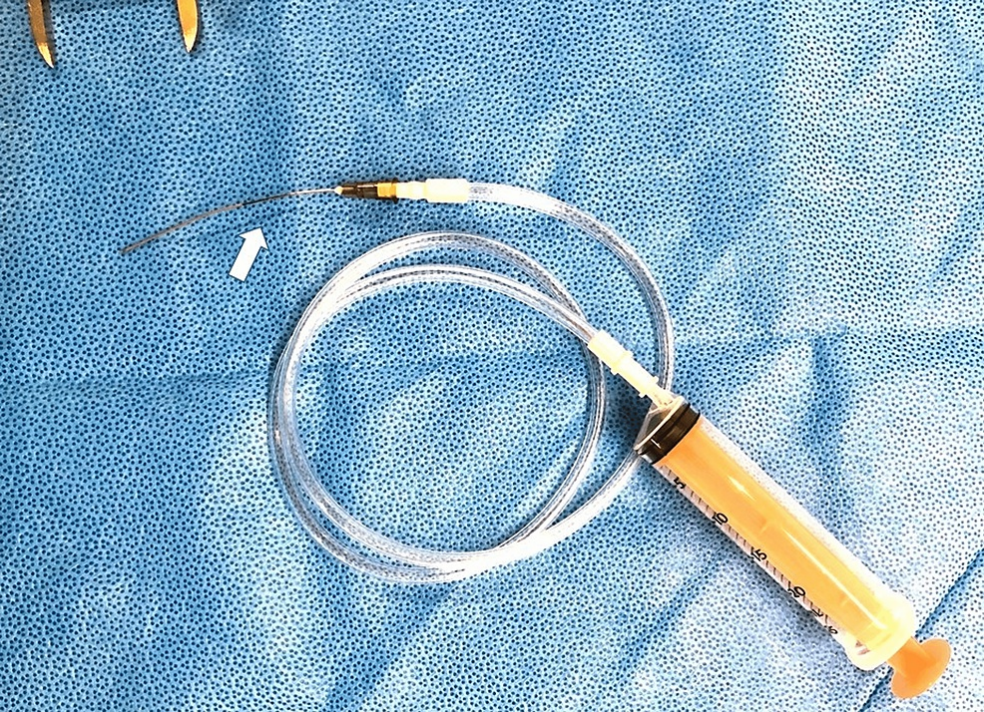
Instrument for FNAC The site 4 cm from the needle tip is marked (arrow). FNAC, fine-needle aspiration cytology.

**Figure 4:**
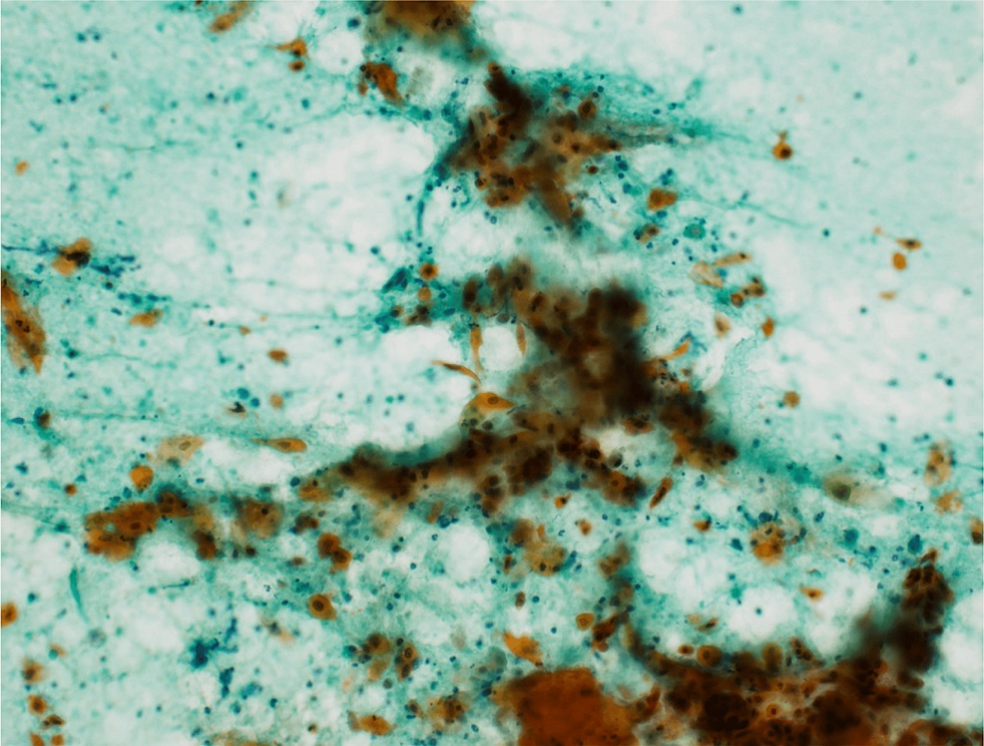
Cytological findings (Papanicolaou stain x400) Numerous ovals to fibrous atypical cells with increased chromatin and orange G-preference are seen against on a necrotic background. Light green preferred oval cells with high nuclear-to-cytoplasmic (N/C) ratio and increased chromatin are also seen in scattered or clustered areas. These findings are consistent with squamous cell carcinoma.

For the FNAC procedure, the nasal surface was anesthetized by applying gauze with adrenaline and 4% lidocaine for 10 min. The patient was seated in an examination chair and the nostrils were dilated using a nasal speculum. FNAC was performed using a device improvised from a 21G catheter needle and an extension tube for infusion connected to a 20 cc syringe (Figure 3). The needle was marked at 4 cm and curved upward, allowing the operator to advance the needle smoothly toward the orbit without penetrating the lacrimal bone or the frontal process of the maxilla. The operator positioned the needle tip, while the assistant applied negative pressure using the syringe. Negative pressure was released after the needle was withdrawn. A pathologist confirmed that an adequate specimen had been collected after the aspirated material was sprayed onto the slide and fixed. A diagnosis of SCC was made on the evaluation of the aspirated material (Figure 4).

Positron emission tomography (PET) and CT revealed no local or distant metastases. The disease was classified as stage IVa (T4aN0M0) [4]. Due to his advanced age, the patient was referred to another hospital for treatment and received proton therapy at a total dose of 60 Gy.

Case 2

**Figure 5:**
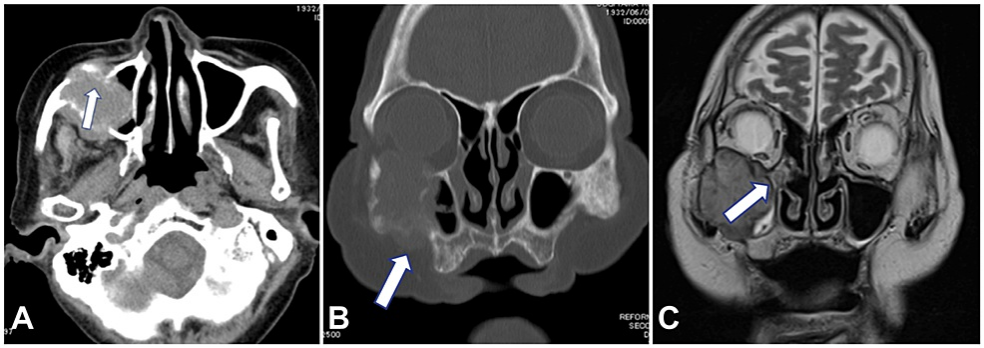
Imaging findings of the paranasal sinuses A) Horizontal section sinus CT. The mass in the right maxillary sinus is infiltrating into the maxillary sinus (arrow). B) Coronal section sinus CT. The mass shadow in the right maxillary sinus is infiltrating outside the maxillary sinus bone (arrow). C) Coronal MRI T2-weighted image. The low-signal mass shadow extends into the right orbit. There is a slightly high-signal shadow in the right middle nasal canal that appears to be secondary inflammatory tissue (arrow). CT, computed tomography; MRI, magnetic resonance imaging.

A 90-year-old man with a history of prostate cancer, cataract, hypertension, and cholecystectomy visited the neurosurgery department with a month-long history of right upper strabismus and diplopia. He was referred to the otorhinolaryngology department because of a mass in the right maxillary sinus. A CT scan of the sinus showed a soft shadow located in the right maxillary sinus, infiltrating into the orbit and the outer maxillary sinus (Figure 5A, 5B). Nasal endoscopy revealed a mass arising from the right middle nasal meatus. A transnasal endoscopic tissue biopsy of this mass was performed in an outpatient room　after local anesthesia was administered in the nasal cavity.

The tissue biopsy showed only inflammatory changes; no neoplastic lesions were detected.

**Figure 6:**
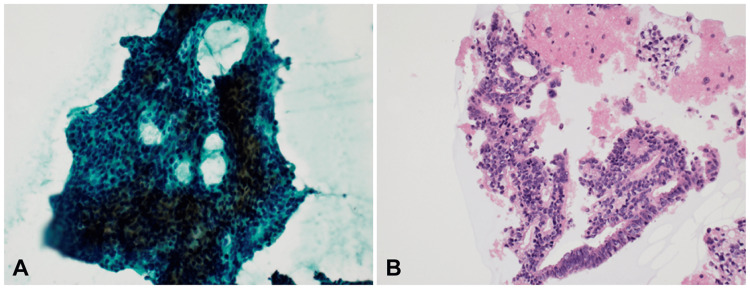
Cytological findings (Papanicolaou stain x200) A) A large number of atypical cells are seen in a solitary to clustered fashion against a background of few inflammatory cells. The atypical cells show nuclear atypia, such as malformed nuclei, increased chromatin, and nuclei of unequal size. There is also a tumor component with pale or foamy cytoplasm, and a mixture of cell clusters with high nuclear-to-cytoplasmic ratio, delicate chromatin, and stacking, and cell clusters with sieve-like structures. B) The cell block specimen shows atypical cells forming an adherent glandular lumen, a finding that is consistent with adenocarcinoma.

For further examination, the patient visited the outpatient clinic of the Department of Otorhinolaryngology, Aichi Medical University three days later. Sinonasal MRI showed a high-signal, iso- to low-signal mass on T1-weighted images and a low-signal mass on T2-weighted images. The mass had extended into the right orbit; a slightly high-signal shadow in the right middle nasal passage suggested a secondary inflammatory tissue (Figure 5C). The mass in the right maxillary sinus breached the anterior wall of the maxillary sinus and extended submucosally. The mucosa anterior to the right maxillary sinus was palpated intraorally and the mass was detected. The right upper lip was then elevated, and FNAC was subsequently performed with an instrument consisting of a 22-gauge catheter needle connected to a 20 cc syringe via an extension tube. The pathologist confirmed that the specimen was adequate. FNAC results showed an adenocarcinoma (ACA) (Figure 6).

He underwent a PET/CT scan, which revealed multiple deposits in the prostate and bone. These were considered metastases from his preexisting prostate cancer. He did not complain of pain or other symptoms apart from diplopia, was elderly, and did not want aggressive treatment. He opted for palliative care. The patient was satisfied with how the definitive diagnosis was promptly established.

## Discussion

The sinonasal tract encompasses the paranasal sinuses (ethmoid, frontal, maxillary, and sphenoid) and the nasal cavities. The paranasal sinuses are in contact with the orbit and the cranium. Primary and metastatic malignancies arising in the paranasal sinuses are rare and histologically diverse. These account for 0.2-0.8% of all malignant neoplasms and 3% of the malignant neoplasms occurring in the head and neck [5]. Primary tumors include SCC, sinonasal undifferentiated carcinoma, ACA, olfactory neuroblastoma, salivary gland type tumors, melanoma, sarcoma, and other rare tumors [3]. Malignant tumors of the sinuses are often overlooked even when initial symptoms (e.g., nasal obstruction, discharge, and bleeding) are present. Patients often report to a hospital after the condition has progressed considerably.

FNAC is an accurate and cost-effective diagnostic method, with high diagnostic accuracy and a short turnaround time. However, because the sinuses are surrounded by bone, tissue biopsy is the mainstay of conventional biopsy. Here, we describe two cases of sinonasal malignancies that were diagnosed using FNAC after tissue biopsies failed to yield a definitive diagnosis. When tumors arise in the nasal sinuses, they often obstruct sinus drainage holes, resulting in secondary sinusitis and inflammatory polyps. Therefore, when a nasal sinus tumor is encountered, imaging (e.g., CT and MRI) should be performed prior to biopsy. Images should be reviewed in three planes.

Sinus CT determines the extent of local invasion into the lamina papyracea, orbital floor, and cribriform and pterygoid plates, and also whether the tumor protrudes outside the sinus cavity into the nasal cavity or skin side. Areas of bone destruction detected on sinus CT are likely to be malignant [6].

Sinus MRI demonstrates invasion of the orbital contents, dura, brain, and cavernous sinuses. It differentiates between normal mucosal thickening and inflammatory tissues such as polyps, which show high signal intensity on T2-weighted images, and neoplastic lesions, which show lower signal intensity on T2-weighted images. In addition, the image pattern of papillomas shows a convoluted cerebriform pattern with alternating high and low signals. Malignant tumors show a heterogeneous internal pattern, making it possible to distinguish areas where benign tumors, such as papillomas, have transformed into malignant tumors [7]. This allows us to differentiate benign tumors, such as papillomas, from those that have transformed into malignant tumors. In case 1, for example, it was possible to identify the portion of the tumor that had undergone malignant transformation.

When a malignant tumor is suspected in the sinus cavity, it is important to image the site of the tumor three-dimensionally by comparing the MRI T2-weighted image with the site of bone destruction on sinus CT.

Combining the information from CT and MRI helps to localize the tumor, identify the biopsy site, and select the appropriate method of biopsy. By marking the site of the tumor on the three-dimensional (3D) CT image for sinus surgery navigation, the corresponding site can be shown on horizontal, sagittal, and coronal CT images on the same screen, making it easier to construct a 3D image of the tumor before biopsy (Figure 2). The direction in which the FNAC needle was to be inserted was confirmed based on the results of the images. If the bone becomes an obstacle, the needle is curved for easier insertion. If the distance from the nasal cavity to the tumor is measured on imaging, the needle is marked at that distance, and is not inserted any further; the cells in the tumor will be aspirated, and the orbit will not be penetrated.

Imaging with contrast, especially with contrast-enhanced CT, can confirm if a lesion is highly vascular [8]. Tissue biopsies of highly vascular tumors are often difficult to perform because of excessive bleeding during the biopsy. However, because FNAC uses a thin needle (21-23G), hemostasis can be achieved by applying pressure using an epinephrine gauze. In elderly patients taking anticoagulants, tissue biopsy under local or general anesthesia is invasive. Prompt diagnosis by FNAC reduces the physical burden on the patients and enables prompt treatment.

Potential puncture sites for FNAC include those where the tumor has invaded extraosseous structures (e.g., subcutaneous, intranasal, or intraorbital invasion from the maxillary or frontal sinuses) or those exposed from the membranous part of the maxillary sinus to the middle nasal passage. Furthermore, FNAC can be performed even for tumors located deep within the ethmoid sinus. Performing FNAC using an intramucosal approach, as in case 2, minimizes the risk of subcutaneous tumor seeding.

Many factors affect the accuracy of FNAC. Experience in performing FNAC is the most important. Failure to obtain a representative sample leads to an erroneous diagnosis. This may be due to a needle positioned outside the target tissue or due to central necrosis, hemorrhage, or cystic change in the tumor [9].

We have created a device composed of a needle, syringe, and an extension tube to facilitate the collection of sufficient samples during FNAC. We also use the same device for ultrasound-guided thyroid cytology; having an assistant apply constant negative pressure prevents needle tip blurring that occurs when the operator performs aspiration; releasing it slowly before removing the needle prevents blood from being aspirated into the needle. The operator should also be aware that when the needle is inserted, it should be rotated concentrically to collect tissue. The pathologist must confirm that enough cells have been collected by examining the fixed aspirated samples on a glass slide. The FNAC is repeated until a sufficient sample is obtained.

According to multicenter reports and meta-analytic studies on FNAC in various organs in the last decade, the sensitivity, specificity, diagnostic accuracy, and area under the receiver operating characteristic curve (AUC) of FNAC for thyroid, lung, breast, and salivary gland tumors, depending on the anatomic site, have been reported to be 85.6%-97.4%, 71.4%-99.6%, and 86.1%-98.5%, respectively (Table 1) [10-13].

**Table 1:** List of recent multicenter or meta-analytic studies reporting sensitivity, specificity, diagnostic accuracy, or AUC of FNAC for thyroid, lung, breast, and salivary gland tumors. 95% CI, 95% confidence interval; AUC, the area under the receiver operating characteristic curve; -, not mentioned in the manuscript; FNAC, fine-needle aspiration cytology.

	Organ	Cases	Sensitivity (95% CI)	Specificity (95% CI)	Accuracy (95% CI)	AUC
Hsiao et al.2022 [10]	Thyroid	16597	85.6% (79.9-89.5%)	71.4% (61.1-79.8%)	-	86.1%
Lee et al. 2019 [11]	Lung	9239	92.5% (91.9-93.1%)	86.5% (85.0-87.9%)	91.1% (90.6-91.7%)	-
de Cursi et al. 2020 [12]	Breast	8334	97.4%	99.6%	98.5%	-
Rossi et al. 2016 [13]	Salivary gland	1729	91.8%	97.6%	91.3%	-

However, there are few reports on the diagnostic accuracy of FNAC for primary nasal sinus malignancies. The following studies reported on FNAC performed on the main body of a nasal sinus malignancy matched with the final histological diagnosis, such as surgical specimens, although the types of cases differed. Helsel et al. performed FNAC on seven nasal sinus tumors and found matches in five cases [4]. Gupta et al. performed FNAC on 64 nasal sinus tumors and found matches in 56 cases [14]. Daskalopoulou et al. found matches in all 21 cases [15]. Reddy et al. reported matches in 73 of 77 cases [16]. The diagnostic accuracies were 71.4%, 87.5%, 100%, and 93%, respectively (Table 2).

**Table 2:** Results of sensitivity, specificity, and accuracy of diagnosis in FNAC of sinonasal malignancies in previous literature reports and our report. FNAC, fine-needle aspiration cytology.

	Cases	Sensitivity (%)	Specificity (%)	Accuracy (%)
Helsel et al. 2003 [A1] [3]	7	85.7	85.7	71.4
Gupta et al. 2011 [A2] [14]	64	90.6	72.7	87.5
Daskalopoulou et al. 1997 [15]	21	100	100	100
Reddy et al. 2015 [16]	77	100	95	93
Our cases, 2023	2	100	100	100

Among these, Dimitra et al. reported the highest diagnostic accuracy; they stated that the diagnostic rate of FNAC can be improved by improving the diagnostic methods, applying stricter protocols in tissue collection, carefully evaluating materials, minimizing procedural limitations, and improving the interpretation of cytological findings in FNAC [15].

SCC is the most common sinonasal tumor, accounting for 40-50% of all nasal sinus malignancies, whereas ACA accounts for 13-19% of all cases [16-18]. FNAC is a good modality for aspirating these tumors, and in the majority of cases, subtyping can also be performed based on cytomorphological features [13]. The cytological diagnosis of carcinoma is relatively easy on Diff-Quik or Papanicolaou-stained material because most carcinomas show cohesive cell groups with cells that exhibit typical epithelial morphology. In general, the cytological features of malignant sinonasal carcinomas are similar to those of carcinomas at other sites. However, immunohistochemical studies are often required for the subclassification of poorly differentiated carcinomas and almost always in all non-epithelial tumors [3].

Although cytology is suitable for detecting individual cellular atypia, it is sometimes difficult to determine structural atypia when cellular stacking is observed [19]. Therefore, a cell block may be prepared. A cell block is a specimen prepared by formalin-fixing and paraffin-embedding the sediment of a cytology specimen. The advantages of cell blocks are that both structural and cellular atypia can be clearly detected, and special stains and immunostains can be added in the same way as for normal formalin-fixed tissue [19]. In case 2, cytology identified a malignant tumor that histopathology could not detect. The image of atypical cells forming an adherent glandular lumen in the cell block was clear, and a clear diagnosis of ACA was made.

An improved diagnostic rate by combining cytological diagnosis and cell block preparations has been reported [19]. The evaluation of FNAC in the diagnosis of the sinonasal area is controversial. However, if FNAC can contribute to the minimally invasive and rapid diagnosis of sinonasal tumors by improving the diagnosis rate through changes in collection methods and specimen preparation, we believe that it can alleviate the mental anxiety of patients that tends to occur before treatment [20].

## Conclusions

FNAC for deep sinus lesions has not been a common diagnostic method for sinonasal tumors because of the difficulty in palpating the tumor and inserting a needle due to the adjacent bone structures. In this paper, we described two cases in which cytology established definitive diagnoses where histopathology had previously failed. Based on these two cases and the literature review, it is important to determine whether FNAC is possible in the paranasal sinus region by performing a thorough imaging diagnosis in advance. If the tumor site can be 3D-reconstructed and cells can be collected from the tumor through FNAC, FNAC is a less invasive, rapid, highly sensitive, and specific test that may be an excellent adjunctive procedure for the diagnosis of nasal sinus tumors.
